# Unusual intrathoracic foreign body: tree branch

**DOI:** 10.1590/0100-3984.2015.0182

**Published:** 2016

**Authors:** Diogo Goulart Corrêa, Tiago Medina Salata, Luiz Sérgio Carvalho Teixeira, Rafael Silveira Borges, Edson Marchiori

**Affiliations:** 1 Universidade Federal do Rio de Janeiro (UFRJ), Rio de Janeiro, RJ, Brazil.; 2 Hospital Casa de Portugal, Rio de Janeiro, RJ, Brazil.

**Figure 1:**
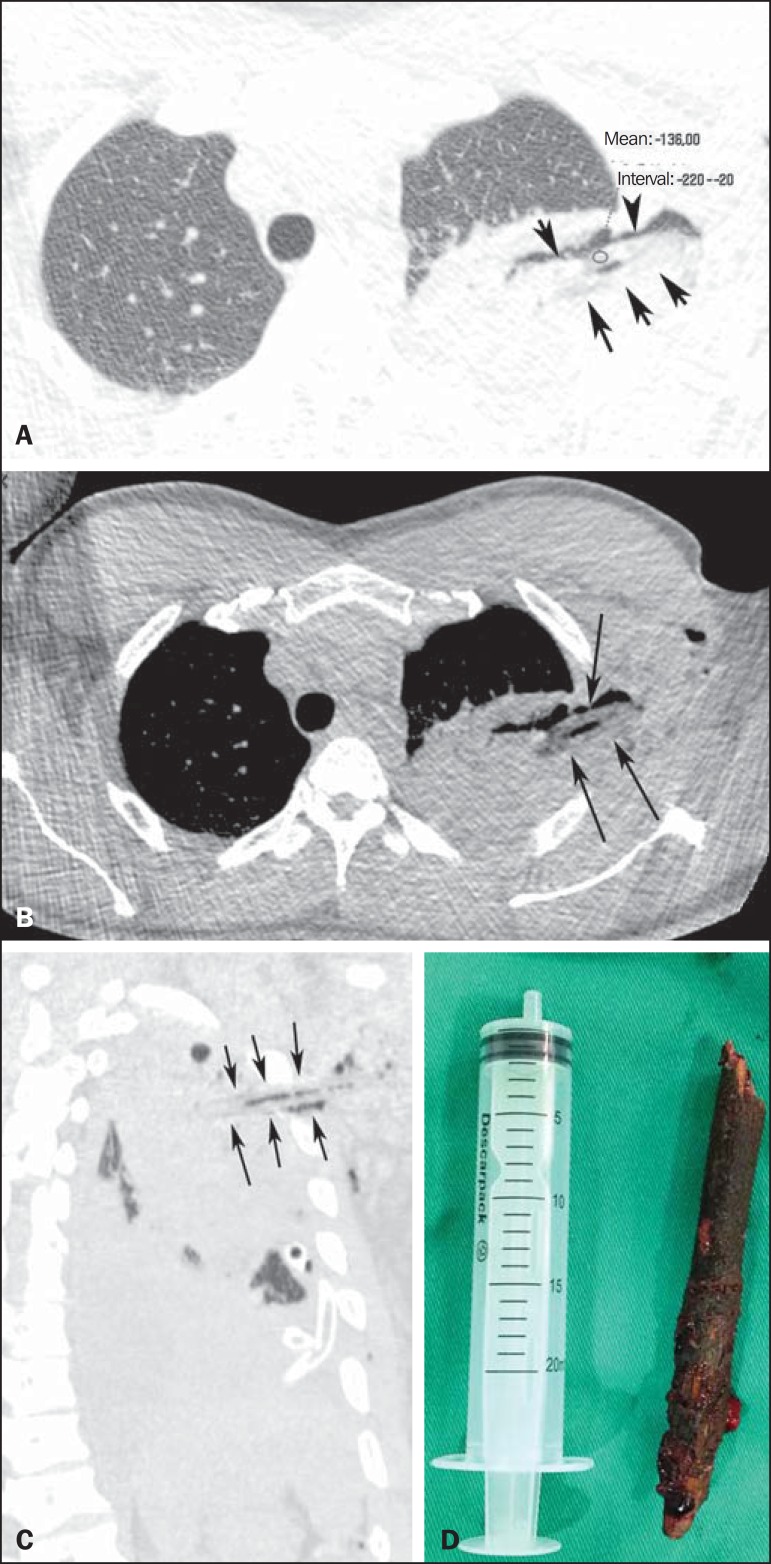
Axial computed tomography scan with a lung window (**A**), intermediate window (**B**), and oblique reconstruction (**C**), showing an elongated foreign body (black arrows) with negative density (-136 HU) and a longitudinal band of air within. Note also the consolidation and aerated areas in the lung parenchyma, which correspond to the contusion and parenchymal laceration. In **D**, the foreign body removed (tree branch).

Dear Editor,

We report the case of a 46-year-old male who was admitted to the emergency room 4 hours after suffering trauma to the left lateral chest wall, which was penetrated by tree branch during a fall from a bicycle. At the time of the examination, the patient was bleeding from the entrance wound and complaining of severe local pain. His vital signs were normal. Computed tomography showed laceration of the left upper lung lobe, with areas of pulmonary contusion and ipsilateral pleural effusion. We also observed a tubular image, with a density of -136 HU, of which the proximal end was in the soft tissues of the chest wall and the distal end was in the lung parenchyma (Figure 1). The patient underwent surgery on the same day, and a piece of tree branch was removed from the chest cavity. A chest tube was inserted, and approximately 1.5 L of blood, mixed with clots, were drained from the pleural space. There was no vascular or mediastinal lesion.

Wooden foreign bodies in the pulmonary parenchyma are rare. Such foreign bodies can penetrate the lungs through open wounds or lacerations in the chest wall^(1)^. When one end of the foreign body is visible on the physical exam, the diagnosis is simple. However, in some cases, the object is not visible externally and the clinical history is unclear. In such cases, the radiological examination is critical to raising the diagnostic suspicion. Unlike foreign bodies composed of metal, bone, glass, or other radiopaque materials^(2,3)^, wooden objects are difficult to identify on radiological examinations. Only approximately 15% of wooden foreign bodies are identified on conventional X-rays^(1)^. Even on computed tomography scans, such foreign bodies can be quite difficult to identify.

The attenuation of a wooden foreign body depends on its porosity and the amount of air and fluid it contains^(1,4,5)^. In general, such objects have negative density, due to the presence of air, and dry wood is less dense than is green wood. In addition, different types of wood have different densities^(4,5)^. In the literature, reported densities range from -24 HU to -656 HU^(6)^. In our patient, the average density was -136 HU. Therefore, the imaging aspect can erroneously suggest gas collections. The differentiation from gas collections can be made on the basis of the shape of the object and the use of intermediate windows in the computed tomography scan. However, wooden foreign bodies, discovered years after their entry, can undergo mineral deposition and become hyperdense^(7)^. Such foreign bodies must be detected and removed as soon as possible because, due to their porosity and organic nature, they constitute an excellent culture medium for microorganisms, which can result in abscesses and fistulas^(1,5)^.

In conclusion, identifying a wooden foreign body can be challenging. The radiologist should keep in mind that wood frequently presents negative density and can in some cases be confused with air collections.
